# AI-Supported Shared Decision-Making (AI-SDM): Conceptual Framework

**DOI:** 10.2196/75866

**Published:** 2025-08-07

**Authors:** Mohammed As'ad, Nawarh Faran, Hala Joharji

**Affiliations:** 1 Corporate Quality & Patient Safety, Dr Sulaiman Al Habib Medical Group, Olaya Street, Riyadh, 12214, Saudi Arabia, 966 920066666; 2 Dr Sulaiman Al Habib Medical Group, Riyadh, Saudi Arabia

**Keywords:** artificial intelligence, shared decision-making, AI reasoning, clinical decision support, generative AI, patient-centered care

## Abstract

Shared decision-making is central to patient-centered care but is often hampered by artificial intelligence (AI) systems that focus on technical transparency rather than delivering context-rich, clinically meaningful reasoning. Although AI explainability methods elucidate how decisions are made, they fall short of addressing the “why” that supports effective patient-clinician dialogue. To bridge this gap, we introduce artificial intelligence–supported shared decision-making (AI-SDM), a conceptual framework designed to integrate AI-based reasoning into shared decision-making to enhance care quality while preserving patient autonomy. AI-SDM is a structured, multimodel framework that synthesizes predictive modeling, evidence-based recommendations, and generative AI techniques to produce adaptive, context-sensitive explanations. The framework distinguishes conventional AI explainability from AI reasoning—prioritizing the generation of tailored, narrative justifications that inform shared decisions. A hypothetical clinical scenario in stroke management is used to illustrate how AI-SDM facilitates an iterative, triadic deliberation process between health care providers, patients, and AI outputs. This integration is intended to transform raw algorithmic data into actionable insights that directly support the decision-making process without supplanting human judgment.

## Introduction

Shared decision-making (SDM) is characterized by collaboration between health care professionals (HCPs) and patients to align with patient values [1]. It has become central to patient-centered care, marking a shift from historical paternalism [2]. Concurrently, artificial intelligence (AI) is increasingly integrated into health care, offering powerful tools for diagnosis, prognostication, and treatment planning [3,4], thereby augmenting clinical capabilities through the analysis of vast datasets [5]. Despite the potential synergies, effectively integrating AI insights into the established SDM process remains a critical challenge.

A key barrier lies in the distinction between artificial intelligence explainability (XAI) and AI reasoning. While XAI focuses on rendering algorithmic processes transparent, primarily for technical validation [6], it often fails to produce justifications that are clinically meaningful and readily communicable within the patient-HCP dialogue. This technical transparency, though important for trust [6], does not equate to the human-centered, contextual reasoning required for SDM. Consequently, there is a disconnect: AI may be explainable technically but not communicable clinically, and traditional SDM frameworks lack mechanisms to incorporate AI-generated reasoning [7].

This paper introduces artificial intelligence-supported shared decision-making (AI-SDM), a conceptual framework designed to bridge this gap. AI-SDM leverages predictive modeling, evidence synthesis, and generative AI to embed AI reasoning, contextual, human-interpretable justifications, directly into the SDM workflow. The framework facilitates collaborative deliberation among HCPs, patients, and AI systems, ensuring AI insights are transparent, contestable, and tailored to individual patient circumstances. By positioning AI as a reasoning facilitator rather than a decision maker, AI-SDM aims to enhance decision quality and evidence-based practice while preserving patient autonomy. Herein, we differentiate AI reasoning from explainability, detail the AI-SDM model and its multimodal AI integration, illustrate its potential application in a clinical scenario, and discuss implementation challenges and future directions.

## AI Reasoning Versus Explainability

Integrating AI effectively into SDM demands clarity on key distinctions between AI transparency, XAI, and AI reasoning. AI transparency provides fundamental visibility into the AI’s process and data, aiming for openness and enabling auditability. This primarily serves regulators, developers, and users needing to understand “What did the system do?”, often via access to code or data flow [6].

Building on this, XAI focuses specifically on illuminating the internal algorithmic logic. Its goal is primarily technical—model validation, debugging, and fairness checks—targeted at developers, data scientists, and auditors’ fairness [6,8]. XAI answers “How did the system produce the output?” using techniques like feature importance scores (Shapley Additive Explanation), heatmaps, or local models (Local Interpretable Model-Agnostic Explanations) [8]. While vital for technical trust and validation [9,10], this technical transparency alone is insufficient for clinical application, as a weight vector or probability score does not equate to a usable explanation for SDM.

AI reasoning, central to the proposed AI-SDM framework, shifts the focus decisively to clinical relevance and justification within the specific patient context. Its goal is to facilitate understanding and deliberation among the key audience: HCPs and patients. It addresses the crucial question, “Why is this output relevant for the patient?” by generating clinically meaningful outputs, such as contextual narratives and risk/benefit summaries, rather than raw algorithmic data [8]. Table 1 summarizes these core distinctions.

**Table 1.** Distinctions among artificial intelligence (AI) transparency, artificial intelligence explainability (XAI), and AI reasoning.

Feature	AI transparency	XAI	AI reasoning (for AI-SDM)^a^
Focus	Visibility of process/data	Internal algorithmic logic	Clinical relevance and justification
Goal	Openness and auditability	Model validation, debugging, and fairness check	Facilitate understanding and deliberation
Audience	Regulators, developers, and users	Developers, data scientists, and auditors	HCP^b^ and patients
Answers	“What did the system do?”	“How did the system produce the output?”	“Why is this output relevant for the patient?”
Example output	Access to code/data flow	Feature importance (SHAP)^c^, heatmaps, LIME^d^	Contextual narrative and risk/benefit summary

a AI-SDM: artificial intelligence–supported shared decision-making.

b HCP: health care professional.

c SHAP: Shapley Additive Explanations.

d LIME: Local Interpretable Model-Agnostic Explanations.

The capacity for AI reasoning has evolved significantly. Historically, clinical decision-making relied on human cognition, later supplemented by early rule-based or probabilistic clinical decision support systems offering limited reasoning capabilities [4,11]. The integration of machine learning and, more recently, advanced large language models (LLMs) has transformed AI’s potential [12-15]. Modern AI can now perform multistep, domain-specific inference [16,17], moving beyond mere pattern recognition to simulate aspects of human deductive, inductive, abductive, and case-based reasoning [18]. AI systems draw on diverse reasoning approaches—from symbolic logic (transparent but less flexible) and statistical methods (probabilistic and less intuitive causality) to opaque neural networks and hybrid neuro-symbolic or knowledge-infused systems aiming for interpretability and semantic alignment [19-21].

This advanced AI reasoning is crucial for SDM, aligning with principles of evidence-based practice and precision medicine [7,22]. SDM requires more than accurate predictions; it demands justifications grounded in clinical workflows, patient history, and anticipated outcomes, enabling deliberation on values and trade-offs [18,23]. AI reasoning provides this by synthesizing large-scale, heterogeneous data (genomic, clinical, real-world evidence) [24] and articulating not just what is predicted, but why it applies to the individual, considering complex risk-benefit profiles and personal priorities [19,21,24-26]. AI reasoning thus acts as a communicative, human-centered layer built upon, but distinct from XAI’s technical foundations [10,23,27]. This distinction reshapes trust: while XAI builds trust via technical validation, AI reasoning fosters interpersonal trust through semantic clarity, contextual relevance, and value alignment within the clinical encounter—prerequisites for meaningful SDM.

## The Role of AI Reasoning in SDM

### SDM as a Process

SDM is a structured yet flexible process in which HCPs and patients collaboratively determine the best course of action, integrating medical evidence with the patient’s values and preferences. Recognizing that many clinical decisions involve multiple valid options, SDM ensures that the chosen path reflects what matters most to an informed patient. The process unfolds in distinct stages [1]. Information exchange serves as the foundation, with HCP presenting viable options, detailing their benefits, risks, and uncertainties. Traditionally, this stage is often supported by static Patient Decision Aids, such as those developed guided by frameworks like the Ottawa Decision Support Framework [28]. The aim is to prepare patients by increasing knowledge and helping clarify values. Deliberation follows, allowing the patient and HCP to explore these options in the context of the patient’s goals, concerns, and circumstances. This phase encourages active dialogue, where patients seek clarification and HCPs ensure comprehension. Decision-making emerges from this discussion, as both parties reach a consensus that aligns clinical expertise with patient priorities. Finally, implementation translates the decision into action, requiring commitment from both patient and HCP. Adherence depends on confidence in the decision, reinforced by clear communication, trust, and continued support through follow-up. While SDM enhances patient engagement and clinical outcomes, its integration into routine practice remains inconsistent. Effective implementation demands a cultural shift in clinical workflows, supported by training, institutional commitment, and tools that facilitate meaningful participation rather than tokenistic involvement.

### Challenges in SDM Addressed by AI and Generative AI

Despite the established benefits of SDM, practical implementation faces substantial barriers that AI, particularly generative AI, can effectively address. The contemporary medical environment presents HCPs and patients with increasingly complex information that can impede effective communication. While traditional AI models provide structured risk stratification and evidence-based recommendations, generative AI complements these by transforming clinical data into adaptive, natural language explanations that facilitate interactive engagement.

A significant barrier is varying health literacy, with many adults struggling to comprehend complex medical information. Generative AI addresses this by converting dense medical reasoning into accessible narratives, calibrated to individual literacy levels through techniques like reading-level adaptation, while preserving clinical accuracy. This supports more meaningful engagement across diverse patient populations without sacrificing informational integrity. Furthermore, AI reasoning can synthesize information related to multiple conditions or comorbidities, presenting a holistic view tailored to the patient’s overall health status, which is often difficult with standard, single-condition PDAs.

Time constraints consistently limit comprehensive SDM implementation. Generative AI streamlines this process by autonomously producing structured, real-time summaries of clinical options and responding dynamically to patient queries. This capability allows HCPs to allocate consultation time to value-based discussions rather than manual data synthesis, enhancing clinical efficiency without compromising decision quality.

Patient heterogeneity in clinical priorities and outcome preferences necessitates personalized communication. Generative AI enables interactive dialogue that adapts to individual concerns. For example, it can restructure treatment comparisons to emphasize nonsurgical alternatives when patients express concerns about operative interventions or highlight specific risks and benefits relevant to the patient’s unique circumstances (eg, comorbidities). This responsive adaptation ensures explanations evolve according to articulated preferences, supporting truly patient-centered communication. To ensure consistency and interoperability, the output generated by AI reasoning systems could be grounded in standardized clinical terminologies, such as Systematized Nomenclature of Medicine Clinical Terms (SNOMED CT). SNOMED CT provides a comprehensive, computer-processable vocabulary for clinical terms used in EHRs globally. Aligning AI-generated explanations with SNOMED CT could help ensure the terminology used is consistent with the patient’s record and potentially compatible with existing structured decision support tools or clinical information systems.

### AI Reasoning Versus Explainability in SDM

In clinical decision support, AI reasoning aims to deliver tailored rationales specific to a patient’s context and values, going beyond technical transparency. Conventional explainability methods, such as feature-importance plots or probability distributions, may reveal how a model arrives at its outputs, yet rarely clarify why a recommendation is meaningful for this patient. By contrast, AI reasoning situates those outputs within clinical logic and patient priorities, generating user-friendly justifications that directly facilitate SDM conversations. In this way, generative AI can transform raw model outputs into narrative explanations relevant to each patient’s unique goals, thus enabling a richer, more interactive exchange than code-level transparency can provide.

The value of AI in SDM lies not in technical transparency but in delivering clear, relevant, and actionable explanations that support informed decision-making. Generative AI enhances this process by enabling real-time refinement of reasoning based on HCP modifications and patient queries. This dynamic responsiveness allows the system to restructure explanations according to evolving priorities, for instance, shifting focus when patients express preferences regarding quality versus length of life, or adjusting the complexity based on literacy needs. Human-level AI reasoning, augmented by generative AI’s capacity to produce adaptive, context-aware explanations, surpasses abstract explainability in clinical relevance and utility, directly supporting the fundamental objectives of SDM in contemporary health care practice.

## The Intersection of AI Reasoning and SDM

### Overlapping Elements of AI Reasoning and SDM

For AI to effectively support SDM, its reasoning processes must align with the communicative and deliberative nature of HCP-patient interactions. Both AI reasoning and SDM inherently demand clarity, transparency, justification, and personalization. For instance, when an AI provides clinically aligned logic, it directly supports the information exchange step by framing recommendations in medical terms that HCPs can relay and discuss with patients. Transparent recommendations facilitate the deliberation phase by clearly presenting options alongside their respective pros and cons. Similarly, a clear justification for AI-generated outputs bolsters the decision-making step, providing concrete, evidence-based rationales. Additionally, AI adaptability to individual patient contexts, values, and literacy levels emulates the tailored communication essential for effective SDM. An AI system capable of communicating through clinical reasoning can seamlessly integrate into the SDM dialogue. In contrast, an AI that provides only raw recommendations without explanations offers limited value in collaborative clinical interactions (Table 2).

**Table 2.** Overlap between artificial intelligence (AI) reasoning components and shared decision-making (SDM) process steps.

AI reasoning component	SDM component	Overlap
Clinically aligned logic	Information exchange	AI must explain decisions in terms of medical reasoning HCPs^a^ can share with patients.
Transparent recommendations	Deliberation	AI reasoning should present options openly, helping patients and doctors compare choices.
Justification of AI outputs	Decision-making	AI should provide clear rationale (“why”) to support the chosen option.
Adaptability to patient context	Tailored communication	AI should adjust its explanations to the individual patient’s needs and values.

a HCP: health care professional.

### What SDM Lacks Without AI Reasoning

When AI reasoning is absent, HCPs and patients are left with raw scores or black-box outputs that fail to address individual preferences and concerns. Moreover, merely disclosing the technical details of a system’s predictions does not sufficiently enable patients to evaluate personal trade-offs. Similarly, it does not help them understand how a recommendation aligns with their health objectives. Consequently, lacking coherent, patient-centered logic, these AI suggestions may appear arbitrary, eroding trust and undermining SDM’s commitment to collaborative, value-sensitive decision-making. Ultimately, advice that lacks contextual reasoning, which both the HCP and patient can discuss meaningfully, turns into top-down instructions. Thus, this approach limits the opportunity for a shared dialogue.

### Bridging AI Reasoning and SDM: Toward AI-SDM

Bridging the gap between AI capabilities and SDM needs requires a shift toward a new paradigm: AI-SDM. This model emphasizes practical integration and technical feasibility in real-world care. AI-generated explanations must be tailored to the clinical context [29,30]. Just as experienced HCPs adjust communication to different scenarios and patient profiles, AI systems should generate context-sensitive justifications. These must reflect clinical reasoning and align with patient values. For example, in chemotherapy decisions, AI reasoning should emphasize expected efficacy based on tumor type, potential side effects, and survival projections—framed according to the patient’s values, such as prioritizing quality of life over longevity. In chronic disease management, such as lifestyle interventions, explanations may instead highlight long-term risk reduction and adherence support. Tailoring AI reasoning to clinical context ensures its explanations are both relevant and usable [30].

Integrating AI-SDM into clinical practice requires alignment with existing health IT infrastructure. A feasible workflow might involve the AI-SDM system being triggered within the electronic health record (EHR) during a patient encounter. The system could leverage modern interoperability standards, such as Health Level Seven International Fast Health care Interoperability Resources application programming interfaces, to interface with the EHR [31]. These standards enable secure retrieval of up-to-date patient data, including diagnoses, medications, lab results, and problem lists coded using SNOMED CT [32]. Predictive AI components would then analyze this data to generate context-specific risk assessments, outcome probabilities, or treatment comparisons based on established models and guidelines. Subsequently, a generative AI component would synthesize these complex outputs into patient-friendly language, creating tailored explanations, summaries, and potentially visual aids. These can be presented directly within the EHR interface for the HCP and patient to review and discuss together.

Successful adoption hinges not only on technical integration but also on stakeholder readiness. Key hurdles include ensuring robust IT infrastructure and establishing privacy-compliant data governance protocols. Providing adequate training for HCPs is also key. This helps them effectively use and critically appraise AI outputs within the SDM context. Strong leadership and organizational commitment are essential to address these challenges, supporting integration and promoting AI as a collaborative tool. This tool enhances, rather than replaces, clinical judgment and patient partnership. Such a standards-based foundation is a prerequisite for reliable data retrieval, consistent interpretation, and effective AI-SDM deployment across diverse clinical settings and platforms.

## Defining AI-SDM: A New Conceptual Model

### Theoretical Foundations of AI-SDM

Dual-process theory of clinical cognition proposes that clinicians alternate between fast, intuitive pattern recognition (system 1) and slower, analytical reasoning (system 2) when diagnosing and selecting treatments [33]. AI-SDM mirrors this architecture by pairing predictive and recommendation models, which emulate System 2’s probabilistic deliberation, with a generative reasoning layer that approximates System 1’s narrative synthesis. This pairing enables the framework to deliver quantitative risk estimates while simultaneously providing context-sensitive justifications that fuel real-time dialogue. The model is further anchored in the Ottawa Decision Support Framework, which conceptualizes SDM as a sequence of need identification, values clarification, and decision support [34]. By embedding adaptive values-clarification prompts within the generative layer, AI-SDM operationalizes these stages and ensures that explanations evolve in response to patient priorities. Principles of patient-centered communication likewise inform system design: explanations are calibrated to individual literacy, emotional state, and cultural context to preserve relational autonomy and encourage bidirectional questioning [35]. Empirical evidence shows that decision aids incorporating tailored narratives and explicit values clarification improve decisional quality and patient trust, particularly when powered by AI-driven reasoning engines that maintain transparency and contestability [36,37]. Synthesizing these theoretical strands positions AI-SDM not merely as a technological overlay but as a cognitive and communicative scaffold that aligns algorithmic inference with the epistemic norms of evidence-based, patient-centered care.

### What Is AI-SDM?

AI-SDM is a comprehensive, multimodel conceptual framework developed to incorporate AI-driven reasoning into clinical decision-making. It explicitly ensures HCP oversight and preserves patient autonomy. Unlike conventional AI-based decision-support tools that often focus solely on algorithmic outputs or technical explainability, AI-SDM introduces a collaborative reasoning approach. It enables real-time interaction and deliberation among HCPs, patients, and AI-generated insights. AI-SDM is built upon a multilayered AI system where different AI models contribute distinct functionalities: predictive AI performs risk stratification and outcome modeling; recommendation AI retrieves evidence-based guidelines and treatment options; natural language processing (NLP) AI extracts relevant data from clinical records; and generative AI functions as the crucial reasoning facilitator, transforming complex, structured AI outputs into interactive, patient-specific explanations. Through this synergistic integration, AI-SDM ensures that AI remains an adaptive and justifiable tool. It allows HCPs and patients to engage in structured deliberation while preserving the core principles of SDM.

AI-SDM builds on advances from sophisticated clinical decision support systems and incorporates Human-Computer Interaction principles for usability. It distinguishes itself fundamentally by its primary goal. That is to generate adaptive, narrative clinical reasoning specifically designed to facilitate the triadic deliberation (HCP-patient-AI) inherent in the SDM process. It shifts the focus from mere prediction or transparency toward context-rich, personalized justifications that clinicians can explore, modify, and communicate in natural language. While Table 1 compared technical forms of AI interpretation, Table 3 expands the comparison to full clinical decision frameworks, contrasting how SDM, XAI, and AI-SDM function at the bedside.

**Table 3.** Comparison of traditional shared decision-making (SDM), artificial intelligence explainability (XAI), and artificial intelligence–supported shared decision-making (AI-SDM) framework.

Dimension	Traditional SDM	XAI	AI-SDM framework (proposed)
Purpose	Aligning decisions with patient values	Explain algorithm outputs	Generate contextual and patient-specific reasoning
Output format	Human dialogue and evidence summaries	SHAP^a^ values, LIME^b^, and saliency maps	Adaptive narrative, visual, and verbal reasoning
Workflow integration	Manual and time-intensive	External to workflow	Embedded within clinical encounter workflow
Personalization	Based on clinician skill/time	Minimal; generalized models	High; tailored to clinical context and patient data
Patient role	Dialogue partner	Passive receiver	Active participant in AI-driven^c^ deliberation
Clinician role	Central guide	Interpreter of AI outputs	Deliberation lead, with modifiable AI input
Use of AI	None	Explanatory only	Multimodel: predictive, generative, NLP^d^, and recommendation
Transparency	Human-led discussion	Technical interpretability	Justifiable clinical reasoning in natural language
Limitations	Time, consistency, and cognitive load	Low usability in clinical conversations	Dependent on quality of AI design and integration
Example scenario	Stroke decision made via verbal counseling	Feature weights for “recommend thrombectomy”	Narrative of options, risks, and priorities generated in-session

a SHAP: Shapley Additive Explanation.

b LIME: Local Interpretable Model-Agnostic Explanations.

c AI: artificial intelligence.

d NLP: natural language processing.

### AI-SDM Workflow and Multimodel AI Integration

**Figure 1.**
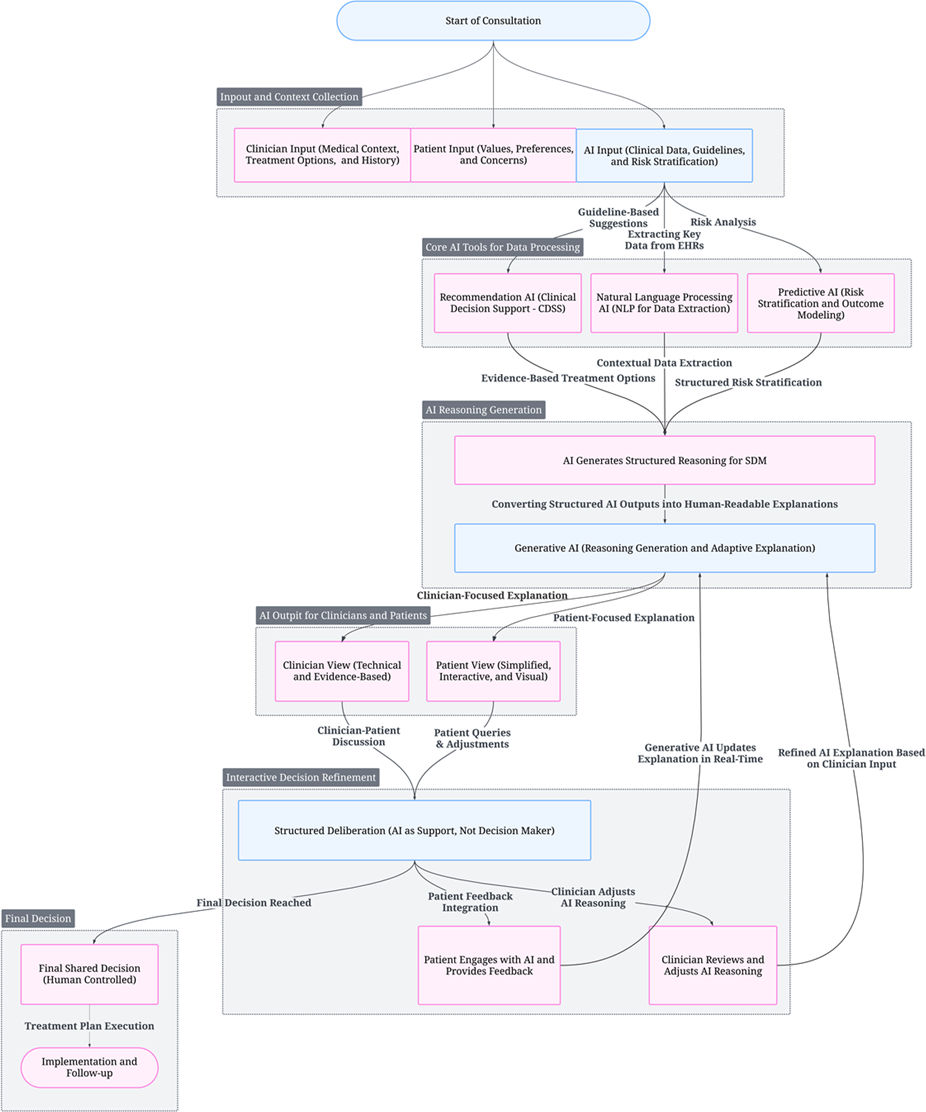
AI-supported SDM conceptual model: a structured, multiphase workflow for integrating AI-generated reasoning into SDM. The model begins with input collection from health care professionals (HCPs; medical context), patients (values, preferences), and AI-derived sources (clinical data and guidelines). Core AI functions, predictive modeling, clinical recommendation, and NLP support contextual risk stratification and evidence synthesis. Generative AI then produces adaptive, human-centered explanations tailored separately for HCPs and patients. The system supports real-time refinement of reasoning through HCP adjustments and patient queries, culminating in a human-controlled shared decision and follow-up planning. Color key: blue boxes within the diagram indicate processes or stages that generate multiple distinct outputs or lead to multiple subsequent steps in the workflow; pink boxes represent processes or outputs that are directly driven or generated by AI components. AI: artificial intelligence; CDSS: clinical decision support system; EHR: electronic health record; NLP: natural language processing; SDM: shared decision-making.

AI-SDM operates through 4 integrated phases. Each phase leverages specialized AI models while preserving HCP oversight and patient autonomy (Figure 1).

The decision process begins with structured data acquisition. This involves gathering information from 3 essential sources: HCP-provided medical history and diagnostic considerations; patient-articulated values, goals, and risk preferences; and AI-derived evidence from clinical guidelines and research findings. During this phase, 3 specific AI functions are used. Predictive AI performs personalized risk assessment and outcomes analysis. Recommendation AI determines evidence-based treatment paths. Additionally, NLP with LLMs extracts unstructured data from health records and literature.

Following data integration, AI-SDM synthesizes statistical models, clinical best practices, and individual patient characteristics into a structured decision model. This model then generates 2 distinct outputs. First, HCPs receive a comprehensive, evidence-based report detailing risk-adjusted treatment pathways, complete with probability estimates and confidence intervals. Second, patients receive an interactive explanation, which may include visual aids, tailored to their understanding. Generative AI plays a crucial role by transforming these structured outputs into context-specific explanations. These explanations are adaptable to user engagement, thereby surpassing the limitations of static AI summaries.

An important conceptual consideration in this multimodel integration is the potential for conflicting or inconsistent outputs. Such conflicts can arise between the predictive, recommendation, and NLP components. For example, a high predicted risk from the predictive model might conflict with a standard guideline recommendation from the recommendation module. To address these conflicts, the AI-SDM framework incorporates a dedicated reconciliation layer. This layer automatically applies a clinically prioritized weighting mechanism. If a conflict occurs, the system assigns greater weight to validated risk factors while flagging any unresolved discrepancies for HCP review. This process ensures full transparency regarding potential ambiguities within the underlying data. Moreover, it maintains a robust foundation that supports subsequent generative AI reasoning. This ensures both transparency and audibility of any data ambiguities.

A central innovation in the AI-SDM workflow involves converting structured algorithmic output into adaptive, human-centered reasoning. Instead of static recommendations, generative AI produces dynamic, context-sensitive explanations that evolve based on HCP and patient interaction. These explanations are explicitly grounded in the underlying evidence and are safeguarded against potential biases or hallucinations (details of this implementation are beyond the scope of this paper). The AI component is designed for adaptability in both content and timing. It can, for instance, provide concise, rapid summaries for acute scenarios or more detailed rationales for planned consultations. The AI delivers reasoning, rather than merely outcomes, through dual channels tailored specifically to HCP and patient needs. This transforms the AI from a data synthesizer into a deliberation partner, supporting more justifiable clinical decisions.

The AI-SDM model facilitates real-time modification of AI-generated reasoning through continuous HCP evaluation and patient engagement. HCPs can adjust recommendations based on their expertise and contextual factors that extend beyond algorithmic reach. Simultaneously, patients can interrogate specific risks and refine their preferences. In response to these inputs, generative AI dynamically updates explanations. This iterative adaptation process ensures continuous alignment with both clinical judgment and evolving patient priorities.

The culmination of this process is a human-controlled, AI-assisted decision that aligns clinical evidence with patient values. AI-enhanced documentation captures the deliberative process, preserving transparency and accountability in medical records. The system can then generate personalized educational materials to support treatment adherence and follow-up strategies, ensuring continuity of care beyond the initial decision point.

### Hypothetical Application: AI-SDM in Stroke Management

#### Overview

The decision to perform mechanical thrombectomy or pursue medical therapy in elderly patients with acute ischemic stroke presents a complex, high-risk clinical scenario requiring rapid yet nuanced deliberation. While thrombectomy significantly improves functional outcomes in patients with large-vessel occlusion, older adults face unique challenges such as increased procedural risks, pre-existing comorbidities, and varied rehabilitation potential [38]. AI-SDM enhances this decision-making process by integrating predictive modeling, evidence-based recommendations, NLP for context extraction, and generative AI to facilitate structured, adaptive reasoning.

#### Scenario

A patient, aged 82 years, presents with an acute ischemic stroke due to an occlusion of the middle cerebral artery. Neuroimaging confirms a substantial penumbral salvageable region with a small infarct core, indicating potential eligibility for thrombectomy based on current criteria [39]. However, the patient has a history of hypertension, mild cognitive impairment, and prior minor strokes, all of which influence the potential for meaningful neurological recovery and postprocedure rehabilitation. The AI-SDM workflow guides the decision-making process by structuring the evaluation into distinct phases, ensuring that clinicians and patients engage in a transparent and data-driven discussion.

#### Phase 1: Input and Context Collection

This phase initiates the process by consolidating patient, clinician, and AI-derived inputs. The clinician provides an assessment of the patient’s neurological status, prestroke function, and imaging results, while the patient and family articulate treatment priorities (eg, maximizing independence) and risk tolerance. AI synthesizes these inputs through distinct subcomponents: predictive AI generates probability-adjusted functional outcome estimates (eg, modified Rankin Scale scores) based on real-world stroke registries and thrombectomy trials [40]; recommendation AI retrieves current stroke management guidelines. Additionally, NLP integrated with LLM extracts relevant historical data from the patient’s records, such as identifying and categorizing symptoms, diagnoses, and treatment plans, which helps clinicians make informed decisions [41]. This comprehensive dataset serves as the foundation for AI-generated reasoning.

#### Phase 2: AI Reasoning Generation

Here, AI-SDM integrates structured insights into a clinical model. It facilitates individualized decision support. The AI synthesizes statistical models predicting outcomes with or without thrombectomy. It incorporates clinical best practices based on guideline recommendations. It also includes patient-specific variables such as age, comorbidities, and imaging findings. These are combined into a structured analysis adapted for clinicians and patient needs. Drawing from studies such as DAWN and DEFUSE-3, the system provides outcome and risk projections [42,43]. It may show based on such studies that thrombectomy increases independence, for example, from 25% to 50%. It may also show a 10% chance of symptomatic intracerebral hemorrhage. The clinician’s view presents a quantitative comparison of 90-day functional outcomes. For patients, generative AI transforms these insights into a simplified, interactive format. It presents recovery trajectories and risks using visual aids and clear language.

#### Phase 3: Interactive Clinician-Patient Deliberation

This phase enables real-time clinician-patient engagement with the AI-generated insights via a dedicated interface supporting both voice and text-based interactions. The patient might query the AI about expected recovery timelines or independence, prompting generative AI to adjust explanations using refined predictive models. The interface simultaneously displays the original and updated outputs side by side, enabling the clinician to review, modify, and discuss these results with the patient. By recalibrating the risk-benefit summary in response to each query, the system keeps every explanation grounded in evidence-based data. This process occurs within a structured deliberation framework where AI is a support, not a decision maker. Because these updates happen in near real time, clinicians and patients remain actively involved in refining the decision until they reach a fully informed consensus. Patient feedback is integrated, and clinicians may review and adjust AI reasoning accordingly. This iterative loop allows both parties to deepen their understanding before reaching a decision.

#### Phase 4: Shared Decision Implementation and Documentation

The process concludes with the clinician and patient reaching a shared decision informed by the AI-assisted deliberation. In this scenario, the patient, having engaged with the structured reasoning, opts for mechanical thrombectomy after weighing the potential benefits against the articulated risks. AI then facilitates implementation by generating structured documentation of the decision rationale for the medical record, ensuring transparency. The shared decision is fully clinician- and patient-controlled, with AI strictly supporting the process. Generative AI can also assist in drafting personalized postprocedure care recommendations, outlining rehabilitation expectations, and follow-up plans. The system continues to support follow-up planning and adaptation, ensuring the implementation remains aligned with patient needs. Throughout, the AI acts as a facilitator, ensuring the decision is guided by evidence and patient values under clinician oversight. Table 4 summarizes these 4 phases using the acute ischemic stroke scenario.

**Table 4.** Summary of artificial intelligence–supported shared decision-making (AI-SDM) phases in the stroke scenario.

AI-SDM phase	Application in acute stroke scenario example
Input and context collection	Patient aged 82 years with MCA^a^ occlusion. Imaging shows salvageable penumbra and small infarct core. Clinician assesses neurological status, prestroke function, and imaging. Patient/family expresses independence goals and risk tolerance. Predictive AI^b^ generates probability-adjusted functional outcome estimates from stroke registries and thrombectomy trials. Recommendation AI retrieves current stroke management guidelines. NLP^c^+LLM^d^ extracts relevant historical data, including symptoms, diagnoses, and treatment plans.
AI reasoning generation	AI integrates predictions, guidelines, and patient variables into structured analysis. Based on DAWN/DEFUSE-3, it estimates outcomes (eg, 25%‐50% independence, 10% hemorrhage risk). Clinician’s view presents a quantitative comparison of 90-day outcomes. Generative AI presents simplified, interactive patient explanations using visual aids and clear language.
Interactive decision refinement	Patient queries recovery timelines or independence. Clinician adjusts AI outputs based on rehab and support. Occurs within a structured deliberation framework where AI is a support tool. Generative AI updates reasoning dynamically. Patient feedback is integrated. Clinicians may review and adjust AI reasoning.
Final decision and implementation	Shared decision made after AI-assisted deliberation. Patient selects thrombectomy. AI documents rationale and generates personalized postprocedure recommendations, including rehab expectations and follow-up. System supports ongoing adaptation. The decision is fully clinician- and patient-controlled.

a MCA: middle cerebral artery.

b AI: artificial intelligence.

c NLP: natural language processing.

d LLM: large language model.

Through this structured AI-SDM approach, complex stroke treatment decisions can remain data-driven, transparent, and patient-centered, leveraging advanced analytics and adaptive explanations within a collaborative framework.

While the stroke scenario illustrates AI-SDM in an acute, time-sensitive setting, the framework’s principles also apply to complex, preference-sensitive decisions in chronic disease management. In advanced chronic kidney disease, particularly among older adults, patients often face substantial burdens and uncertain benefits from dialysis and may remain uninformed about conservative kidney management as a treatment choice [44,45]. Likewise, in cardiology, decisions such as whether to pursue left atrial appendage occlusion instead of long-term anticoagulation for atrial fibrillation, or how to manage advanced heart failure in line with patient goals, frequently require nuanced SDM discussions [46]. AI-SDM can help address these challenges by integrating longitudinal data, evidence-based predictions, and patient-reported outcomes, thus facilitating more individualized deliberation around what matters most to each patient over the course of their illness trajectory.

### Ensuring AI-SDM Preserves Patient Autonomy

A fundamental requirement for AI-SDM is that it must safeguard patient autonomy and uphold the ethos of SDM at every step. To this end, the model is designed such that the AI’s outputs are always transparent, open to question, and subordinate to human input. Both the HCP and the patient should be empowered to challenge or adjust the AI’s suggestions freely. For example, if the AI’s analysis seems to favor a particular treatment strongly, the patient can ask for clarification or express discomfort, and the HCP can probe the AI’s reasoning for validity—in both cases, the AI must accommodate these challenges by explaining its rationale or recalibrating its advice. This contestability is deliberate: the AI is not a black box oracle handing down decisions, but a tool that invites scrutiny. Transparency is crucial here; the AI-SDM system should clearly communicate why it is highlighting certain options (eg, “Option A is supported by X study for patients with your profile”) so that the human participants can critically evaluate the reasoning. By avoiding opaque or one-sided recommendations, the AI prevents any undue influence or bias that could pressure the patient. In practice, this means AI-SDM will present multiple options with evidence rather than a singular “do this” directive, and it will explicitly incorporate the patient’s own goals into its analysis. The HCP retains ultimate responsibility to interpret and, if necessary, correct the AI’s output before any action. In sum, AI-SDM is constructed as a facilitator, not a decision maker: it expands the information and reasoning available to the patient and HCP, but it never replaces their agency. The patient’s values and the HCP’s professional judgment remain at the center of every decision, thereby preserving the autonomy and individualized nature of care.

## Challenges and Future Directions

The successful integration of AI into SDM requires proactively addressing critical implementation barriers to ensure clinical uptake, effectiveness, and ethical deployment.

### HCP Adoption and Trust

Adoption hinges on transparent, interpretable AI systems that avoid “black box” functionality. In AI-SDM, generative AI transforms complex algorithmic outputs into verifiable explanations with clear references to clinical guidelines and explicit confidence levels. Implementation requires structured reasoning pathways that allow HCPs to interrogate AI-derived conclusions and understand their evidentiary basis, particularly when recommendations diverge from conventional practice.

### Regulatory Landscape and Liability

Navigating the evolving regulatory frameworks for AI-assisted clinical decision-making is crucial. AI-SDM systems, particularly those providing diagnostic or therapeutic recommendations, would likely be considered software as a medical device and need to align with guidelines from regulatory bodies like the US Food and Drug Administration or equivalent authorities globally. Key considerations include rigorous validation, demonstrating safety and effectiveness, ensuring transparency (allowing HCPs to independently review the basis for recommendations), and implementing robust quality management systems, including postmarket surveillance. While AI-SDM preserves human oversight by positioning AI as decision support rather than the ultimate decision maker, clear governance policies are needed to delineate responsibility among developers, HCPs, and health care institutions, especially concerning liability if AI suggestions deviate from the standard of care.

### Ethical Considerations and Equity

AI-SDM must be implemented ethically, safeguarding patient rights and promoting equity. This includes strict adherence to data privacy regulations pertinent to health information, such as the principles outlined in the Health Insurance Portability and Accountability Act in the United States or the General Data Protection Regulation in Europe, as well as relevant national or local regulations (eg, in Saudi Arabia). Systems must be designed to accommodate diverse health literacy levels, cultural contexts, and cognitive abilities, and generative AI interfaces should dynamically adjust explanation complexity based on individual needs while preserving clinical accuracy. Furthermore, proactive measures are essential to address potential algorithmic biases, which could arise from training data used in the predictive or recommendation models. To prevent AI hallucinations and preserve clinical integrity, each generative output is anchored to explicit citations from validated guidelines or peer-reviewed studies. An automated audit protocol continuously monitors real-time outputs for discrepancies, flagging any deviations from established evidence standards so that HCPs can rapidly override or adjust the AI’s recommendations. This includes rigorous auditing of the underlying predictive and recommendation models for fairness across demographic groups and designing the generative AI reasoning layer to explicitly surface significant uncertainties or conflicting evidence that might stem from data limitations or potential biases.

Building on these safeguards, future deployments will institute a 4-layer governance loop for continuous bias mitigation. First, training pipelines will use fairness-aware algorithms—such as reweighting and equalized-odds postprocessing—to correct calibration disparities before clinical deployment, an approach recommended by Rajkomar et al [47] for advancing health equity in machine-learning systems. Second, the production environment will stream model outputs into a real-time dashboard that audits performance by age, sex, ethnicity, and socioeconomic status; similar bias-auditing infrastructures have been shown to reveal hidden performance gaps in widely used clinical algorithms [48]. Third, quarterly ethical-compliance reviews will examine data provenance, feature attribution, and workflow impact to maintain regulatory alignment, and finally, all bias metrics and remediation actions will be logged in a version-controlled registry to support external audit and public transparency. Together, these stages create an auditable feedback loop that limits drift, documents remediation, and embeds fairness governance directly into routine system maintenance.

The sociotechnical impact of AI-SDM also depends on how clinicians and patients adopt, negotiate, and contest its recommendations. Rogers’ Diffusion of Innovations theory explains variability in uptake by highlighting perceived complexity, relative advantage, and trialability, whereas technological-determinist perspectives warn that overly authoritative AI may erode clinician agency, and social-constructivist analyses emphasize that users actively reshape technology through practice [49]. To preserve balanced doctor-patient dynamics, AI-SDM therefore labels the scope and limitations of every recommendation, requires explicit clinician confirmation before any automated action, and provides a “why-question” interface so both parties can interrogate underlying evidence or override suggestions. Empirical work on person-centered AI indicates that transparent, assistive designs strengthen trust when clinicians retain control, while unmoderated reliance can attenuate empathy and SDM [50]. Embedding these sociological insights into interface rules and governance policies anchors AI-SDM in relational autonomy and guards against power imbalances.

### Technical Integration and Workflow

The clinical utility of AI-SDM depends on seamless integration with existing EHR systems and clinical workflows. Implementation requires user-friendly interfaces that generate concise, contextually relevant insights without increasing cognitive burden or documentation requirements for HCPs. However, seamlessly embedding this potentially complex, multistep interaction, particularly the deliberative refinement phase, into time-constrained and varied clinical workflows represents a significant practical and design hurdle. Achieving this without disrupting clinical practice or unduly lengthening consultations will be critical for successful adoption. As discussed earlier (in section “Bridging AI Reasoning and SDM: Toward AI-SDM”), leveraging interoperability standards like Fast Health care Interoperability Resources and terminologies like SNOMED CT is vital. Ultimately, AI-SDM must demonstrate measurable improvements in decision quality, patient experience, or efficiency to justify the technological investment and workflow adjustments required for widespread adoption.

Addressing these multifaceted challenges necessitates an iterative implementation approach, combining continuous HCP and patient feedback with rigorous validation. Validating the efficacy and safety of the AI-SDM framework itself would require a phased approach, progressing from algorithmic validation of individual AI components and rigorous usability testing of the interface and explanation formats, through simulation studies assessing decision quality, to eventual pilot clinical trials evaluating real-world impacts on patient engagement, decision concordance, and outcomes. Successful deployment will ultimately depend on collaborative governance structures that balance technological innovation with clinical pragmatism, ethical principles, patient safety, and regulatory compliance.

### Future Directions

Realizing the potential of AI-SDM necessitates substantial future research and development. Key priorities include the rigorous development and refinement of the generative reasoning component, incorporating robust mechanisms for clinical validity, grounding, and bias mitigation, alongside effective strategies for reconciling outputs from disparate AI models. While this paper introduces AI-SDM as a conceptual framework, future work could explore empirical validation, such as usability studies, workflow simulations, or clinical implementation pilots, to assess its impact on decision quality, patient engagement, and workflow integration. Further research grounded in HCI principles may also inform how the model could integrate seamlessly into clinical environments without increasing HCP burden. Finally, ongoing investigation into dynamic fairness auditing, evolving regulatory pathways for AI-driven SDM tools, and establishing clear governance structures will be crucial for responsible and equitable deployment.

Building on the dual-process and patient-centered theories outlined above, future iterations of AI-SDM will deepen its affective intelligence by coupling multimodal emotion‐recognition pipelines with the existing generative explanation layer. Recent work demonstrates that equipping decision-support systems with emotional capabilities can reduce affective bias and improve user trust when complex trade-offs are discussed [51]. To operationalize this insight, we plan to integrate a multimodal deep learning model that fuses facial microexpressions, vocal prosody, and lexical sentiment—an approach shown to outperform unimodal affect detectors in health care contexts and to strengthen access trust between patients and clinicians [52]. Continuous emotion streams will inform dynamic values-clarification prompts generated by the narrative engine, ensuring that explanations adapt when signs of confusion, anxiety, or decisional conflict emerge. A recent systematic review of emotion-recognition AI identifies transparent feature attribution and dataset diversity as prerequisites for reliable affective computing in clinical environments; these requirements will guide our data-governance and model-validation strategy [52]. Finally, evidence from a randomized trial of an AI-enabled decision aid shows that personalized, empathetic narratives significantly improve decisional quality and shared-decision metrics compared with static educational material. By embedding such adaptive affective feedback into AI-SDM, we not only enhance the emotional-computing module but also further align the framework with the Ottawa Decision Support and patient-centered communication theories that underpin its interdisciplinary foundation.

## Conclusions

Integrating AI into clinical practice requires more than predictive accuracy; it demands alignment with patient-centered care principles like SDM. This paper introduces AI-SDM, a conceptual framework designed to bridge this gap. AI-SDM leverages predictive modeling, evidence synthesis, and generative AI to embed AI reasoning, contextual, human-interpretable justifications, directly into the SDM workflow, facilitating collaborative deliberation among HCPs, patients, and AI, ensuring insights are tailored. However, several limitations warrant attention, including the need for pilot studies to test real-world feasibility, clear protocols for reconciling conflicting model outputs, and safeguards against AI hallucinations. Immediate next steps will involve simulation-based validation and user-centered design iterations to refine how AI-SDM integrates with existing clinical workflows. While significant implementation challenges remain, including ethical considerations, regulatory alignment, and workflow integration, AI-SDM offers a promising pathway. By synergizing AI’s analytical power with the personalized approach of SDM, this model can potentially enhance decision quality, foster patient autonomy, and advance evidence-based, patient-centered care in the era of intelligent health systems.

## Abbreviations:

AI: artificial intelligence
AI-SDM: artificial intelligence–supported shared decision-making
EHR: electronic health record
HCP: health care professional
LLM: large language model
NLP: natural language processing
SDM: shared decision-making
SNOMED CT: Systematized Nomenclature of Medicine Clinical Terms
XAI: artificial intelligence explainability
